# A Test for Carbolic Acid

**Published:** 1874-04

**Authors:** 


### A TEST FOR CARBOLIC ACID.

Plugge has observed that when a solution containing carbolic acid is mixed with a solution of the subnitrate of mercury, not only is the mercury reduced, but the liquid acquires an intense red color. Further experiments showed that the presence of traces of nitrous acid was essential to this reaction, and that it could be employed as a test for carbolic acid. He found that the color was still quite evident when only one sixty-thousandth part of carbolic acid was present. Still smaller quantities could be detected by this test if carefully applied, but the amount of nitrous acid must be very small. —*Jour. of App. Chem.*

